# Emotional and Physical Health Impact in Children and Adolescents and Their Caregivers Using Open-source Automated Insulin Delivery: Qualitative Analysis of Lived Experiences

**DOI:** 10.2196/37120

**Published:** 2022-07-14

**Authors:** Katarina Braune, Niklas Krug, Christine Knoll, Hanne Ballhausen, Axel Thieffry, Yanbing Chen, Shane O'Donnell, Klemens Raile, Bryan Cleal

**Affiliations:** 1 Department of Paediatric Endocrinology and Diabetes Charité - Universitätsmedizin Berlin Berlin Germany; 2 Berlin Institute of Health Berlin Germany; 3 Institute of Medical Informatics Charité - Universitätsmedizin Berlin Berlin Germany; 4 School of Sociology University College Dublin Dublin Ireland; 5 #dedoc° Diabetes Online Community Berlin Germany; 6 Jay Keasling Faculty, BioInnovation Institute Center for Biosustainability Technical University of Denmark Copenhagen Denmark; 7 Intomics A/S Kongens Lyngby Denmark; 8 School of Public Health, Physiotherapy & Sports Science University College Dublin Belfield Ireland; 9 Diabetes Management Research Steno Diabetes Center Copenhagen Herlev Denmark

**Keywords:** automated insulin delivery, closed-loop, do-it-yourself, open source, peer support, patient-reported outcomes, lived experiences, qualitative analysis, mobile phone

## Abstract

**Background:**

Given the limitations in the access and license status of commercially developed automated insulin delivery (AID) systems, open-source AID systems are becoming increasingly popular among people with diabetes, including children and adolescents.

**Objective:**

This study aimed to investigate the lived experiences and physical and emotional health implications of children and their caregivers following the initiation of open-source AID, their perceived challenges, and sources of support, which have not been explored in the existing literature.

**Methods:**

Data were collected through 2 sets of open-ended questions from a web-based multinational survey of 60 families from 16 countries. The narratives were thematically analyzed, and a coding framework was identified through iterative alignment.

**Results:**

A range of emotions and improvements in quality of life and physical health were reported, as open-source AID enabled families to shift their focus away from diabetes therapy. Caregivers were less worried about hypoglycemia at night and outside their family homes, leading to increased autonomy for the child. Simultaneously, the glycemic outcomes and sleep quality of both the children and caregivers improved. Nonetheless, the acquisition of suitable hardware and technical setup could be challenging. The #WeAreNotWaiting community was the primary source of practical and emotional support.

**Conclusions:**

Our findings show the benefits and transformative impact of open-source AID and peer support on children with diabetes and their caregivers and families, where commercial AID systems are not available or suitable. Further efforts are required to improve the effectiveness and usability and facilitate access for children with diabetes, worldwide, to benefit from this innovative treatment.

**International Registered Report Identifier (IRRID):**

RR2-10.2196/15368

## Introduction

### Background

Type 1 diabetes (T1D) is a challenging chronic condition for children, adolescents, and their caregivers and is associated with long-term macro- and microvascular complications and the consequent risk of increased morbidity and mortality. Therapeutic guidelines of the International Society for Pediatric and Adolescent Diabetes recommend a target hemoglobin A_1c_ (HbA_1c_) level of <7% for children and adolescents with T1D, albeit a target that must be balanced with the individual disease burden and risk of hypoglycemia [1].

The management of diabetes is particularly challenging during childhood and adolescence. Day-to-day tasks often involve an entire family. Children show variability in insulin sensitivity related to physical growth and sexual maturation, which requires frequent adjustments in insulin dosing [2]. With the dynamic physical activity and nutritional intake of young children, their glycemic levels can fluctuate rapidly [3]. In addition, the transition of responsibility for diabetes management from caregivers to children and their increasing independence during adolescence can often further complicate this difficult dynamic. Adolescents and young adults with diabetes frequently struggle to meet the recommended glycemic targets and are particularly vulnerable to acute complications, such as severe hypoglycemia and diabetic ketoacidosis [4,5]. Living with T1D also impacts the quality of life and mental health [6]. Thus, psychosocial support and individualized treatment play an important role in diabetes care in this age group [1].

Recent advances in diabetes technology have led to the development of automated insulin delivery (AID) systems, also known as hybrid closed-loop, closed-loop, or artificial pancreas systems. In AID, a control algorithm automatically adjusts the insulin delivery of an insulin pump in response to readings from a continuous glucose monitor (CGM) to help improve glycemic levels and variability and reduce the day-to-day burden in diabetes management [7-10].

Although commercially developed AID systems have recently become available in select countries, not all are licensed for use by children. Currently, CamAPS FX (CamDiab Ltd) is the only AID system that has received regulatory approval for children aged ≤7 years but is restricted in interoperability and only compatible with one specific CGM and pump model, only available in select European countries, and must be individually purchased on a subscription basis. Young children are often the last cohort to be included in a clinical trial. Off-label use of commercial AID in this group shows only minor time in range (TIR) and HbA_1c_ improvements compared with older individuals, indicating a higher hypoglycemia risk for this age group [11,12].

Parents and caregivers of children with diabetes have been in the vanguard of the drive toward AID systems. Under the hashtag *#WeAreNotWaiting*, a web-based patient community has sought to create resources and tools for diabetes management since 2013. The movement began with the “Nightscout” project, where caregivers created a cloud-based platform for alerts and remote glucose monitoring for their children. Eventually, the community developed control algorithms for the AID. In these “do-it-yourself” or “open-source” AID systems, commercially available sensors for CGM and insulin pumps are linked to a microcontroller or an app on a smartphone. The source code and documentation of these systems were shared freely on the web. In addition, the community provides both practical and emotional peer support with setup and maintenance. To date, open-source AID systems have not been approved by regulatory bodies and must be built and used at an individual risk. An estimated number of >10,000 individuals, worldwide, use open-source AID. Approximately 20% of these users are children and adolescents, where their caregivers are building and maintaining the systems on their behalf [13,14].

Although evidence based on the clinical outcomes of open-source AID is growing, there are relatively few published studies on the lived experiences of people with diabetes using this technology and fewer still, concerning children and adolescents with diabetes and their caregivers. Previous studies have found improvements in HbA_1c_ and percentage TIR in various age groups, including children and adolescents [13-16]. As part of the Outcomes of Patients’ Evidence with Novel, Do-it-Yourself Artificial Pancreas Technology (OPEN) [17] project, we previously assessed self- or caregiver-reported clinical outcomes [14,15] and motivations [14] to build open-source AID. Improved sleep quality was a primary reason for caregivers to use AID, followed by improved glycemia and reduced complication risk for the child, and the option of remote monitoring and control via the internet, thus reducing disease burden and enabling more independence for children. A recently published international consensus statement on open-source AID supported its use for children and adolescents, as long as the child's welfare is being considered by health care professionals (HCPs) and caregivers who are setting up open-source AID systems for their children, with the child's assent and engagement [18].

### Objectives

This study aimed to examine four specific, albeit interrelated, aspects of the lived experiences of children and adolescents with diabetes and their caregivers on their journey to becoming open-source AID users: (1) the emotional health implications of open-source AID, (2) the experience of changes to physical health using open-source AID, (3) perceived challenges with the implementation and maintenance of open-source AID, and (4) sources of support during the implementation and maintenance of open-source AID. Self-reported glycemic outcomes and sleep have also been reported to provide further context for lived experience data.

## Methods

The results were obtained from answers to 2 open-ended questions included in a cross-sectional web-based survey examining the use of open-source AID. The survey titled “DIWHY” was conducted between November 2018 and March 2019 [17].

### Ethics Approval

Ethics approval was provided by Charité—Universitätsmedizin Berlin (EA2/140/18).

### Survey Design

The survey (Multimedia Appendix 1) was created by the patient-led OPEN consortium in collaboration with further users of open-source AID and was piloted with a small group before the final release. The Checklist for Reporting Results of Internet E-Surveys (CHERRIES) guideline was used to guide the survey development [19]. The survey included 39 items in total, including questions on the child’s demographic information, the open-source AID system in use, HbA_1c_, and TIR before and after initiation, and 2 composite open-ended questions, which sought to capture lived experiences with open-source AID in the form of narratives. Participants could enter a free-text answer with up to 1000 words for each of the 2 questions.

The first question inquired about the individual journey of the caregiver and child toward setting up and using an open-source AID system, including sources of information, support, motivation, and emotional impact:

If you would like, please share your personal story about why you decided to build your own artificial pancreas system and how you got started. Feel free to share any experiences that had a significant impact on how you manage your diabetes as well. This story can be as short or as long as you wish.

The second question addressed the perceived changes following the initiation of open-source AID and the challenges experienced:

When reflecting on your personal DIY closed-loop story, you may want to consider the following: When did you first hear about DIY closed-loop systems and how did you look for further information? Were there any key events or experiences that were a factor in your decision to begin closed looping? Was there anyone else involved in helping you come to decision to begin DIY closed-looping? For example a friend, family member or an online support group? What were your emotions in the lead-up to building your DIY closed-loop system? For example, had you any major hopes or fears?

### Participants and Recruitment

Participants were eligible if they were caregivers of a child or adolescent with diabetes (type 1, 2, or other), using an open-source AID. There were no restrictions on age, time since diagnosis, or commencement of open-source AID.

Participants were recruited using public announcements on the OPEN project website and social media channels, such as the Facebook groups “Looped” (>6000 members) and “AndroidAPS Users” (>1800 members as of November 2018), regional subgroups, and tweets under the hashtags *#WeAreNotWaiting* and *#DIYAPS*. All posts were organic, meaning that their web-based reach was not affected by any monetary influence. Participants consented electronically and joined voluntarily and anonymously with the children’s assent. The survey was available in German and English.

### Data Collection and Analysis

Data were collected and managed using the REDCap (Research Electronic Data Capture; Vanderbilt University) electronic data capture tools hosted at Charité. Following deidentification of the data set, qualitative analysis was performed using NVivo 12 (QSR International, 2018) software. The narratives were analyzed using an approach based on the principles of Template Analysis [20]. Acknowledging the response priming included in the framing of the open-ended questions, initial coding (by KB, CK, and NK) sorted the data in accordance with 4 predefined topics: physical health impact, emotional impact, sources of support, and perceived challenges. To establish alignment, all 3 coders analyzed and sorted the first 30 narratives into 4 topics. Using the “coding comparison” function in NVivo, it was established that there was a high level of agreement among the coders. Level of agreement was defined as the number of units of agreement divided by the total units of measure within the data item, as a percentage. In the next phase of data analysis, all data extracts sorted into the 4 topics were coded inductively and independently by the 3 coders, which led to an extensive set of descriptive codes. Finally, codes were collaboratively collated and used to establish a set of higher-level codes, each of which was described in detail in a codebook. The template codebook was refined and modified in discussions between the 3 coders and the project group.

To test the utility and resonance of the themes as captured in the template, 2 coders (HB and BC) further used the template to analyze the narratives independently of one another. The initial group of coders (KB, CK, and NK) then refined the template based on the coding and feedback provided during this process. A third independent coder (SO) then analyzed the data using the refined template. After this final review of coded responses and the template, it was agreed that code saturation had been achieved, and all major themes were identified.

Retrospective and caregiver-reported clinical outcome data were analyzed within the R (R Foundation for Statistical Computing) programming framework. Only respondents who reported at least one value before and after open-source AID commencement were considered, leading to sample sizes of N=52 and N=36 for HbA_1c_ and TIR, respectively. The HbA_1c_ values were averaged. Moreover, 1-tailed Student *t*tests were conducted with the parameter *paired=TRUE*. Figures were produced using the ggplot2 package**.**

## Results

### Participant Characteristics

In total, 60 caregivers (35.7% of all 168 participants in the DIWHY study) from 16 countries responded to the open-ended questions on behalf of their children, and there were combined 107 responses to both questions. All children and adolescents were diagnosed with T1D, aged between 3 and 20 years, and using an open-source AID for a duration of <1 month and up to 3 years. The caregiver and child demographics as well as the clinical features of the 60 participants who responded to the open-ended questions are summarized in Table 1, whereas the characteristics of the other 108 participants of the DIWHY study are included in Table S1 in Multimedia Appendix 2.

**Figure 1:**
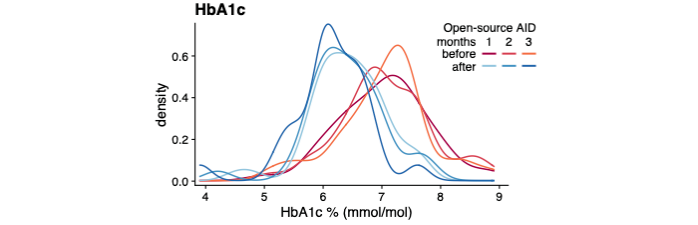
Outcomes of open-source automated insulin delivery (AID) implementation. Density distributions of hemoglobin A1c (HbA1c) before and after commencement of open-source AID (line colors); n=52.

**Figure 2:**
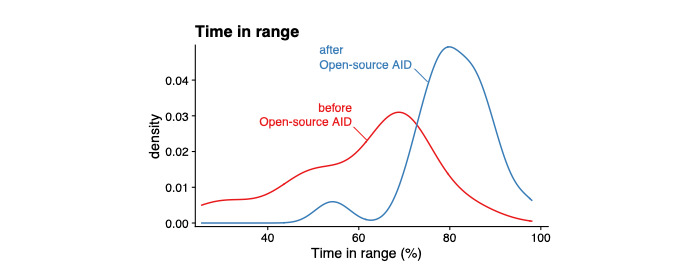
Outcomes of open-source automated insulin delivery (AID) implementation. Density distributions of time in range (70-180 mg/dL/3.9-10.0 mmol/L) before and after commencement of open-source AID (line colors); n=36.

Of the 60 children and adolescents, the average HbA_1c_ levels (of participants with reported measures both before and after AID commencement, see Methods section) decreased from 7.0% (SD 0.8; 53 mmol/mol) to 6.3% (SD 0.7; 45 mmol/mol; 1-tailed paired *t* test; *P*<.001; Figure 1), and TIR increased from 60.7% (SD 15.1) to 80.4% (SD 9.1; 1-tailed paired *t* test; *P*<.001) following the initiation of open-source AID (Figure 2).

**Table 1:** Children’s and caregivers’ demographic and self-reported clinical characteristics (N=60).

				Children and adolescents
**Child’s gender, n (%)**				
	Female		26 (43)	
	Male		34 (57)	
	Other		0 (0)	
Child’s age (years), mean (SD)				10.0 (4.5)
**Type of diabetes, n (%)**				
	Type 1		60 (100)	
	Type 2		0 (0)	
	Other		0 (0)	
Duration of diabetes (years), mean (SD)				5.3 (4.3)
Duration of open-source AID^a^ use (months), mean (SD)				10.9 (9.2)
**Type of open-source AID, n (%)**				
	AndroidAPS		28 (47)	
	OpenAPS		21 (35)	
	Loop		17 (28)	
	Other^b^		2 (3)	
**Region and country of residence, n (%)**				
	**Europe**		47 (78)	
		Germany	12 (20)	
		United Kingdom	9 (15)	
		Finland	7 (12)	
		Sweden	5 (8)	
		Czech Republic	3 (5)	
		Spain	3 (5)	
		Slovakia	3 (5)	
		Others^c^	14 (12)	
	**North America**		6 (10)	
		United States	4 (7)	
		Canada	2 (3)	
	**Asia**			
		South Korea	3 (5)	
	**Western Pacific**			
		Australia	5 (8)	
**Caregiver’s education: highest completed**, **n (%)**				
	University degree or diploma		38 (63)	
	Doctorate		9 (15)	
	High school		8 (13)	
	Other		5 (8)	
**Caregiver’s occupational status, n (%)**				
	Full-time		39 (65)	
	Part-time		15 (25)	
	Unemployed		3 (5)	
	Other		3 (5)	
**Annual household income (US $), n (%)**				
	<20,000		4 (7)	
	20,000 to 34,999		5 (8)	
	35,000 to 49,999		4 (7)	
	50,000 to 74,999		11 (18)	
	75,000 to 99,999		10 (17)	
	>100,000		16 (27)	
	I would rather not say		3 (3)	
	Not stated		7 (12)	

^a^AID: automated insulin delivery.

^b^“Open loop with AndroidAPS” and “custom development.”

^c^Austria, Bulgaria, Croatia, and Greece.

### Template Analysis

#### Overview

A total of 4 topics, “Emotional and Quality of Life Impact,” “Physical Health Impact,” “Challenges,” and “Support” were used to organize the qualitative data, recognizing the fact that participants’ responses were partially primed by the framing of the open-ended questions. The data were subsequently analyzed to generate codes within these topics to expand and illustrate them. The codes are described with examples of illustrative quotes, the number of occurrences, and the respondents’ profiles, as shown in Table 2.

**Table 2:** Final codebook template including deductively (A-D) and inductively (A1-D2) developed codes.

Topic		Occurrences^a^, n	Illustrative quote	Respondent profile
**Emotional and quality of life impact (A)**				
	Worry and fear (A1): describes difficult emotions such as worry and fear of caregivers related to living with and managing diabetes, experiencing hypoglycemia, and developing long-term complications. It also refers to the concern of not being able to build and maintain the open-source AID^b^.	16	“I was very skeptical and scared. Over time more information became available and the safety became clear and compelling. We realized we would be safer with a Loop than without. I was scared that others would not be able to comprehend this (because even endocrinologists fail to understand fully the burden and dynamism of type 1) and that they would question whether we were putting our child at risk and make a report about us to child well-being authorities.”	Caregiver of a boy aged 13 years, from Australia; aged 6 years at diagnosis; using Loop for 2.5 years
	Desperation and frustration (A2): describes feelings of desperation and frustration of caregivers related to living with diabetes and caring for a child with diabetes, diabetes management, and the implementation of the open-source AID.	14	“As a mom I was desperate, I was tired from being up all night, I was getting frustrated from teen hormones and I was willing to try almost anything to help both of us.”	Caregiver of a girl aged 17 years, from the United States; aged 2 years at diagnosis; using Loop for 33 months
	Uncertainty (A3): describes uncertainty and insecurities of caregivers regarding legal grounds, missing regulatory guidelines, and the trust of reliability in an open-source AID system.	6	“Nevertheless, there is still a legal uncertainty and at the moment we just dare to use the loop in our own four walls. In the morning we switch to the normal AnyDana A app, in the evening back to AndroidAPS.”	Caregiver of a boy aged 12 years, from Germany; aged 11 years at diagnosis; using OpenAPS for 2 weeks
	Anticipation, hope, and wishes (A4): describes positive and hopeful emotional states of anticipation and great expectations of caregivers that lie on the AID for improved diabetes management and hope for improved quality of life. Also includes wishes for access to an AID system for everyone.	24	“Major driver for the project was to give my son more years without complications by lowering the HbA_1c_^c^.”	Caregiver of a boy aged 18 years, from Finland; aged 1 year at diagnosis; using OpenAPS for 1 year
	Excitement, appreciation, and satisfaction (A5): describes all positive emotions of caregivers and children related to the experience with the open-source AID in daily use including excitement, happiness, satisfaction with the results, and appreciation.	51	“I remember the exact place I stood watching the [OpenAPS] log [roll] and seeing the [preflight] was successful and then that the loop was complete. I was in shock that we could do this and that I could afford it and that my child was going to [be] better off because of this. It was a defining moment in my life as a parent. No one could stop me giving my child the care they needed anymore. Especially not a company who places shareholders above clients (which legally they must do). I was no longer at the mercy of markets, profits, politics and whims, I had the capacity to provide for my child again.”	Caregiver of a boy aged 13 years, from Australia; aged 6 years at diagnosis; using Loop for 2.5 years
	Security and reassurance (A6): relates to caregivers feeling more empowered, more secure, and reassured owing to the use of an open-source AID system, through automation, remote monitoring, and control, as well as experiencing success and observing the success of others using an open-source AID.	45	“Our child never woke up if she had a low even though her pump was sounding a very loud alarm. And because she slept in her own room we were afraid of sometimes not hearing the pump alarm either. So Nightscout sounded like the perfect solution, as we could then be woken up by any mobile phone or iPad. This added a lot to our feeling of security.”	Caregiver of a girl aged 10 years, from Finland; aged 7 years at diagnosis; using OpenAPS for 3 months
	Child empowerment and independence (A7): describes the degree of independence, autonomy, and self-determination in children and adolescents using the open-source AID, enabling them to participate in daily life and social activities in a responsible and self-determined way.	25	“Daughter can work without having to phone me for advice. She has been on holiday [for the] first time without parents. She[...] now feels confident to consider leaving home.”	Caregiver of a girl aged 20 years, from Croatia; aged 10 years at diagnosis; using OpenAPS for 3 months
**Physical health impact (B)**				
	Glycemic outcome improvement (B1): refers to improved time in range and HbA_1c_ levels, less glucose variability, fewer hypo- and hyperglycemic events, and reduced long-term complication risk.	36	“Every single morning she’s in range. If at night she’s not, we know that by the morning she will be, and she [will get] there safely. It’s really good.”	Caregiver of a girl aged 18 years, from the United Kingdom; aged 11 years at diagnosis; using AndroidAPS for 8 months
	Quality of life improvement (B2): refers to the mentioned improvements of quality of life and describes the degree to which an individual is healthy, comfortable, and able to participate in or enjoy life events.	14	“I keep a continuous discussion with my twins that both use DIY closed loops, through texting. I use this way to share my remote observations on their status, while they concentrate on their university studies, or simply enjoy their lives. I inform them this way about a failing connection, a reservoir getting empty, a battery needing charging, or to drink some juice to avoid a coming low.”	Caregiver of a boy, aged 20 years, from Greece; aged 2 years at diagnosis; using OpenAPS for 1 year
	Improved sleep (B3): denotes all aspects of improved sleep quality for either caregivers or children such as increased sleep duration, fewer sleep interruptions, and feeling better rested in the morning.	40	“It’s been as good as expected, and better still as now we sleep. You forget how much sleep deprivation clouds your judgment.”	Caregiver of a boy aged 8 years, from the United Kingdom; aged 7 years at diagnosis; using AndroidAPS for 3 months
	Facilitated diabetes management (B4): relates to the simplifications of the individual diabetes management due to the open-source AID, such as fewer interactions with the technology or between caregiver and child; for example, through remote control and automation. It also includes the age-appropriate transfer of responsibilities from caregivers to adolescents to self-manage diabetes therapy.	42	“There is no comparison with earlier. There used to be 5-6 blood measurements per child per day, and that was all. With or without a pump, every meal was a challenge. For 1.5 years, the APS has been adjusting the blood sugar value after the bolus, adding more insulin if the value increases, or adjusting the delivery if the value drops.”	Caregiver of a boy aged 20 years, from Greece; aged 1 year at diagnosis; using OpenAPS for 17 months
**Challenges (C)**				
	Access to technology (C1): relates to the issue concerning obtaining access to the component parts of an open-source AID system, such as loopable pumps and supplies, CGM^d^, and additional required hardware.	27	“Getting the hardware was most frustrating. I tried to buy the hardware from the manufacturer but in Sweden you could not do that without a subscription from your doctor. I ended up getting a second hand Dana R pump from another patient who upgraded to a newer pump.”	Caregiver of a boy aged 3.5 years, from Sweden; aged 2 years at diagnosis; using AndroidAPS for 4 months
	Out-of-pocket expenses (C2): describes barriers regarding out-of-pocket expenses and cost for the hardware and supplies related to insurance coverage, household income, and other financial challenges in access.	6	“We were concerned about the cost of sensors. They are not covered by private health here and it cost approximately US $5000 a year when we started. Now kids are covered, but when they turn 21 that ends. We are still worried about covering that bill in the future.”	Caregiver of a boy aged 13 years, from Australia; aged 6 years at diagnosis; using Loop for 2.5 years
	Self-perceived lack of technical skills (C3): denotes the issue of yet self-perceived limited knowledge and missing technical skills caregivers are experiencing to set up open-source AID initially.	9	“Major fears I wouldn't be able to understand the technology.”	Caregiver of a girl aged 12 years, from Australia; aged 11 years at diagnosis; using Loop for 1 month
	Lacking health care provider support (C4): relates to instances where caregivers reflect upon their children’s health care providers’ lack of support and negative attitudes.	14	“Fight with our own diabetologist to get a DANA RS prescribed. Although we didn’t talk openly about looping, she has repeatedly emphasized that we only want the DANA RS pump for looping, which is not allowed. We have won, but now hide the loop, which cannot be a permanent state. We need medical care in which we can communicate openly.”	Caregiver of a boy aged 12 years, from Germany; aged 11 years at diagnosis; using AndroidAPS for 2 weeks
	Impracticability of carrying additional devices (C5): relates to the necessity for children and adolescents having to carry additional devices for open-source AID and protect them from breaking.	9	“It also meant that our daughter had to carry an extra item, i.e. the mini-computer, with her during the day.”	Caregiver of a girl aged 10 years, from Finland; aged 4 years at diagnosis; using OpenAPS for 2 months
	Transition from childhood to adulthood (C6): describes challenges associated with the transition from childhood to adulthood, regarding physical and hormone-related changes during puberty and psychosocial challenges in adolescents living with T1D^e^ and taking over responsibility for their own therapy with an open-source AID.	10	“While our control has improved, it is still significantly more variable than I would expect based on the results I see from others in the community. My son is highly insulin sensitive [...], variable in his activity level and intensity [...], and experiencing substantial swings in carb ratios, basal rates, and insulin sensitivities as he is going through great physiological changes in puberty.”	Caregiver of a boy aged 11 years, from the United States; aged 8 years at diagnosis; using OpenAPS for 1 year
	Setup and maintenance effort (C7): relates to difficulties caregivers experience while setting up open-source AID. This includes an unexpected high time effort and multiple throwbacks while initially setting up the system, technical difficulties with running and maintaining the system, and fine-tuning to find the right settings and parameters to accomplish desired results.	54	“As a family, we feel very happy that we can finally control the blood sugar levels of our children in the desired area, even if it takes great care to do everything right. Batteries (pump, CGM, mobile phone, OpenAPS computer) must be regularly charged or exchanged, the CGM must be continuously calibrated, insulin must be refilled, every 3 days you exchange the catheter, every 14 days the CGM, etc. With such a result, no problem. The hundreds of hours I've spent on it are worth it.”	Caregiver of a boy aged 20 years, from Greece; aged 1 year at diagnosis; using OpenAPS for 17 months
**Support (D)**				
	Community peer support (D1): includes actively received or provided community peer support. This support could either be provided on the web through social media groups and communities or in person through life events, individual people, or meet-ups. Does not include individual key people or role models.	45	“So in that same Facebook group I started to learn about DIY artificial pa[n]creases and I joined another, international group called Looped to learn more. I then asked around and I was told that OpenAPS was the most advanced of the three options and decided to go for that.”	Caregiver of a girl aged 10 years, from Finland; aged 7 years at diagnosis; using OpenAPS for 9 months
	Individuals as role models (D2): describes one or multiple key people, often members of the #WeAreNotWaiting community, who inspired or directly supported caregivers and children in building an open-source AID.	15	“I found Tim Street’s Diabettech website and started following him on twitter/blog at [the] same time. He was coming to speak at a medical conference in Edinburgh and was going to a [type 1] meet up. I gate-crashed the meet in the pub and had to wait until the end[...] I asked him to show me his pancreas! [...] Tim organized the first U.K. meet up in London and offered me an old transmitter which would complete my build. My son and I flew to London and we got going that evening.”	Caregiver of a boy aged 12 years, from the United Kingdom; aged 8 years at diagnosis; using OpenAPS
	Web-based resources (D3): describes web-based resources such as wiki blogs, tutorials, websites, webinars, and other documentation.	19	“Once I had the equipment, I set the system up in two nights, the instructions available on the web are very clear and I found it easier tha[n] expected.”	Caregiver of a girl aged 10 years, from Finland aged 6 years at diagnosis; using OpenAPS for 3 months
	Health care professionals (D4): this code refers to the support provided by health care professionals, such as pediatricians, endocrinologists, and other members of the diabetes teams, including help with setup, access to components, and fine-tuning of settings.	8	“Endocrinologist was supportive even though legally couldn’t recommend it.”	Caregiver of a girl aged 12 years, from Australia; aged 11 years at diagnosis; using Loop for 1 month

^a^Defined by the number of codes assigned to a text segment.

^b^AID: automated insulin delivery.

^c^HbA_1c_: hemoglobin A_1c_.

^d^CGM: continuous glucose monitor.

^e^T1D: type 1 diabetes.

#### Topic 1: Emotional and Quality of Life Impact

For respondents, experiences with the initiation of open-source AID were associated with a range of emotions, from worry, despair, and great hopes before use, to excitement, relief, and a feeling of empowerment after implementing the system. Caregivers in the sample expressed concerns when opting to choose an open-source AID, but it also highlights the deep-rooted frustration and dissatisfaction with commercially available solutions for diabetes management. Therefore, choosing to opt for an open-source AID was never a decision taken lightly but at the point when all other options appeared inadequate and insufficient.

Once the choice was made, quality of life improvements and reductions in the burden of diabetes management were frequently mentioned. With the automation of insulin delivery, families could reboot everyday life without diabetes management being constantly the center of attention, empowering children and caregivers to experience more freedom and flexibility:

Now we plan for things in our lives. We have been thinking of getting a pet, [and have] started to remodel our house. [We] made sure both kids have passports because now it feels like we actually can travel and show them the world.

Caregiver of boy aged 8 years, from Sweden; aged 1 year at diagnosis; using OpenAPS and AndroidAPS for 1.5 years

The option to remotely follow and control glycemic levels, treatments, and insulin delivery via Nightscout reassured caregivers was specifically mentioned as a reason to choose open-source AID. Caregivers experienced fewer worries about their children experiencing hypoglycemia at night or away from home, which led to greater independence, empowerment, and age-appropriate participation of children in their own treatment.

The complexity of the implementation process of open-source AID raised concerns among some of the respondents, who were initially worried about not being able to manage the technical setup on their own. Uncertainties regarding the safety of new and unfamiliar therapies have also been mentioned. Furthermore, they were unsure whether the new treatment would be accepted by their children’s health care team as well as their wider social environment. In addition, some expressed the need for regulatory approval and improved access to AID for everyone:

I wonder how it can be that such a development is not already established? Why does it take so long? Do the old systems have to be remunerated? The loopers show how it works, how can it be that with so much added value, the professional institutions are still so lethargic?

Caregiver of a boy, from Germany, aged 1 year at diagnosis; using Loop for 3 months

Overall, caregivers described the transition to open-source AID as a predominantly positive experience for the entire family. They were highly satisfied with the outcomes and benefits for their children’s emotional and physical health and perceived open-source AID as the best therapy option available:

If I could give my pancreas to my son, I would. This is the next best available option.

Caregiver of a boy aged 12 years, from the United Kingdom; aged 3 years at diagnosis; using Loop for 1 month

#### Topic 2: Physical Health Impact

Improvements in glycemia, such as improvements in HbA_1c_ and TIR levels, as well as less hypoglycemia and fewer glucose fluctuations, have been extensively described:

Every single morning she's in range. If at night she's not we know that by the morning she will be, and she [will get] there safely.

Caregiver of a girl aged 18 years, from the United Kingdom; aged 11 years at diagnosis; using AndroidAPS for 8 months

In addition to diabetes-related health improvements, better sleep quality was frequently highlighted by the respondents. Before using an open-source AID, many caregivers were not able to sleep through the night as they were concerned with nighttime hypoglycemia or the administration of correction doses of insulin, poor sleep, and reduced quality of life. With an open-source AID, they were released from frequent check-ups and the associated emotional pressure:

We were waking at 11 pm, 2 am, 5 am, etc to manually [blood glucose] check our daughter. We haven't done that in years. I was having seizures from almost 5 years of not sleeping more than a couple [of] hours at [a] time. Now, we all sleep all night.

Caregiver of a girl aged 8 years, from the United States; aged 4 years at diagnosis; using Loop

Even in cases with little improvement in glycemic outcomes, where HbA_1c_ and TIR levels were in or close to the recommended targets before the initiation of open-source AID, caregivers noted that the amount of effort required to achieve these results was significantly diminished. As this point highlights, the data repeatedly pointed to the ways in which physical outcomes are inextricably intertwined with emotional outcomes when considering diabetes management.

#### Topic 3: Challenges

Difficulties in accessing compatible hardware have frequently been reported. This was mainly associated with differences in the availability of insulin pumps and sensors and reimbursement policies among countries and also with out-of-pocket expenses. Some participants raised concerns regarding access to components and financial aspects of maintaining their open-source AID system in the future:

We were concerned about the cost of sensors. They are not covered by private health here and it cost approximately US[D] 5000 a year when we started. Now, kids are covered, but when they turn 21 that ends. We are still worried about covering that bill in the future.

Caregiver of a boy aged 13 years, from Australia; aged 6 years at diagnosis; using Loop for 2.5 years

Understanding the documentation and initial setup process is time consuming and challenging, especially for caregivers with little pre-existing knowledge in technology. Ultimately, the complex setup procedure led to a better understanding of the functionalities of open-source AID, enabling caregivers to better respond to technical issues when they occurred. Being part of the *#WeAreNotWaiting* community, caregivers felt gratitude for the available peer support and resources to help with the technical and practical aspects.

Once the setup was successfully managed, the next perceived challenge was the iterative determination of the appropriate settings and therapy parameters to generate satisfactory results. This “fine-tuning” was described as requiring considerable time and endurance. The need to carry around additional devices (eg, a microcontroller or bridge device to remotely communicate between the phone and insulin pump) poses further practical challenges for children in daily life.

The attitudes of HCPs involved in diabetes care of children were described as mixed, ranging from proactive support to refusal:

After detailed research, the reserved position of our center could not stop us either. In the past year, I have repeatedly had the impression of knowing more about the disease and the possible forms of therapy than the doctors at our center.

Caregivers of a girl aged 10 years, from Germany; aged 6 years at diagnosis; using Loop for 1 year

Despite these reported clinical and quality of life improvements, some expressed uncertainty arising from a lack of support from health care providers. Consequently, a family decided not to disclose the use of open-source AID to their health care team, which caused feelings of isolation, disappointment, and misunderstanding:

Although we didn't talk openly about looping, [our diabetologist] has repeatedly emphasized that we only want the DANA RS pump for looping. [...] We[...] now hide the loop, which cannot be a permanent state. We need medical care in which we can communicate openly.

Caregiver of a boy aged 12 years, from Germany; aged 11 years of age at diagnosis; using AndroidAPS for 2 weeks

#### Topic 4: Sources of Support

Participants frequently approached the *#WeAreNotWaiting* community for their support. Social media groups play a key role, where many users share their experiences, respond to questions, discuss related topics, and provide peer support. These were also sources of reassurance in cases of concerns or uncertainties. The extent and quality of peer support available was often a key factor in their decision-making, establishing a sense of trust in the systems, even in the absence of health care provider support or regulatory approval:

So in that same Facebook group I started to learn about DIY artificial pa[n]creases and I joined another, international group called Looped to learn more. I then asked around and I was told that OpenAPS was the most advanced of the 3 options and decided to go for that.

Caregiver of a girl aged 10 years, from Finland; aged 7 years at diagnosis; using OpenAPS for 9 months

Besides the peer support caregivers found on the web, they attended in-person meetings and local meet-ups with members of the community. Lectures, workshops, and public presentations of open-source AID developers, researchers, and other users and parents enhanced their motivation to start their own journey toward open-source AID. Key individuals who were integral in the development of open-source AID are personally named on a number of occasions. The perceived integrity and altruism of these individuals were also key in creating a sense of confidence and trust in the systems:

I found Tim Street's Diabettech website and started following him on Twitter [...]. He was coming to speak at a medical conference in Edinburgh and was going to a [type 1] meet up. I gate-crashed the meet in the pub and had to wait until the end[...] I asked him to show me his pancreas! [...] My son and I flew to London and we got going that evening.

Caregiver of a boy aged 12 years, from the United Kingdom; aged 8 years at diagnosis; using OpenAPS

Although HCPs could not prescribe open-source AID systems owing to the absence of regulatory approvals, some were very supportive of the children’s and caregivers’ decision to use open-source AID. Support by HCPs has mostly been reported regarding access to compatible components, such as specific insulin pumps and CGM types that are prescriptible. In a small number of cases, individual caregivers reported that their health care provider initiated a discussion about open-source AID and directed them to relevant sources of information. Conversely, a lack of support from HCPs was also articulated in a number of accounts, although this usually took the form of “turning a blind eye” and passivity, and very few reported being actively opposed by their health care provider. Where such cases did occur, it usually took the form of preventing caregivers from acquiring the hardware needed to set up an open-source AID system.

## Discussion

### Principal Findings

In this qualitative analysis, we described the emotional and physical health impact of open-source AID use in children or adolescents and their caregivers, as well as their perceived challenges and sources of support.

Overall, caregivers reported a range of emotions before and after the initiation of open-source AID use. Before initiation, for example, participants reported frustration and dissatisfaction with their existing diabetes management solutions and anticipation and excitement—sometimes marked with anxiety and trepidation—at the prospect of using an open-source AID. Likewise, the experience of using open-source AID evoked both great joy and relief, but this was also tinged, for some, with frustration and worry. Improvements in children’s diabetes management, glycemic outcomes, physical health beyond diabetes, and emotional well-being were highlighted in the narratives. Furthermore, sleep quality and quality of life improved for both children and caregivers. The initial challenges were difficulties in accessing the required components, lack of confidence in technical skills for setup and maintenance, concerns about the response from health care teams, and the wider social environment of the family. Later, finding and “fine-tuning” of the right therapy settings, as well as the impracticality of carrying additional devices for the children, were described. The *#WeAreNotWaiting* web-based community was frequently approached as the primary source of information as well as emotional and practical support.

This study can inform stakeholders regarding the unmet needs of children and adolescents with T1D regarding the therapeutic options available to them. Furthermore, our findings highlight how children might benefit from customizable open-source AID systems where commercial options are not accessible, approved for certain age groups, or limited in their functionality to cover the lower and variable insulin requirements of children.

### Comparison With Prior Work

The ethical and legal aspects of the off-label use of unregulated medical devices in children and adolescents are multifaceted and complex. Although the off-label use of pharmaceuticals is both common practice and a necessity in pediatrics, it is still uncommon in medical devices. HCPs were sometimes perceived to be reticent in their support of the decision to use open-source AID. This reticence is understandable given that many HCPs, as indicated by the caregivers in this study, had very little knowledge of the systems and uncertainty regarding what legal ramifications there might be in providing support for a system not approved by regulatory bodies. Following a number of position papers from several local diabetes organizations, a group of international HCPs recently provided an international consensus statement for practical guidance on the safe and ethical use of open-source in clinical settings [18]. The consensus encourages colleagues to learn about all treatment options that could help people with diabetes, including open-source AID, and to support individual decisions to use open-source AID for treatment, as long as benefits and risks are understood. In addition, children’s welfare must always be considered by caregivers and HCPs, with their assent and engagement [18].

Although there are numerous studies about the clinical outcomes of the use of open-source and commercially available AID systems in adults and children [13-15,22-24], there is yet very limited knowledge about the lived experiences and psychological antecedents or consequences leading to the use of and with AID. To the best of our knowledge, this is the most extensive study on lived experiences in children and adolescents using open-source AID, and their caregivers and families, conducted at a multinational level. Our findings are in line with other studies that indicated a reduced burden of diabetes in users of commercial and open-source AID [8,25-31]. Caregivers’ sleep and mental and physical health in the context of their children’s diabetes remains an underresearched area. A reduced burden on caregivers of young children was previously identified as the main outcome of the use of a commercial AID system [8]. The DIWHY survey was conducted between 2018 and 2019. At the time, only one commercially developed AID system was approved and made available in the United States. We did not explicitly ask for this information, although with only 4 participants from the United States, it can be assumed that most of the participants did not have access to commercial AID. Furthermore, open-source AID systems have continuously improved over time with respect to usability and device interoperability. For example, the need to carry around additional hardware may no longer be applied in the recent versions of AndroidAPS, FreeAPS, and Loop. We suggest further research in this field to provide a better understanding of the full psychosocial and economic impact of any kind of AID, as well as the challenges in the access and use of these systems.

### Strengths and Limitations

This study has several strengths and limitations. Of particular strength is its patient and public involvement in the study design process and its multinational scope. Limitations include that the anonymous study design did not allow participants to follow up for clarification or further questions, to strictly follow the General Data Protection Regulation guidelines. A selection bias may be present with the survey only being available in German and English, which may have excluded users not proficient in these languages in the first place. Furthermore, those within the sample might not have responded in detail, or not at all, to the open-ended questions owing to language barriers among other factors. In addition, a significant proportion of the OPEN team was German, with strong links to the German diabetes community; therefore, the teams’ ability to reach people was particularly high in that country. Finally, the challenges in building and setting up an open-source AID had to be overcome by caregivers with perseverance and self-motivation in the first place, potentially resulting in a selected population that limits broad generalizations to all people with diabetes.

### Conclusions

With frequent changes in insulin requirements, glycemic variability due to counterregulatory hormones, and physical activity, children are ideal candidates for AID. Although the uptake of insulin pumps and CGM is high among children in countries where access to diabetes technology is facilitated, the uptake of AID in children is protracted owing to the license status of commercially available AID systems. However, their efficacy in young children and those with low insulin requirements remains limited. Furthermore, glycemic outcome improvements in the off-label use of commercial AID systems by very young children are suboptimal, although they experience similar glycemic improvements as older children, adolescents, and adults with commercial systems approved for their age [7] and with open-source AID [13-16]. Our findings indicate a transformative impact of open-source AID in children and adolescents of various ages on their emotional and physical health, as well as their and their caregivers’ sleep and quality of life. They further highlight how remote monitoring and control are perceived by parents to be safe and how the children are provided with greater autonomy.

Similar to commercial AID systems, there remains much room for improvement in open-source AID systems, and further research is needed to improve the effectiveness of algorithms and usability of AID systems in general, particularly in young children where approved therapy options remain limited. To achieve this, concerted efforts are required using a multi-stakeholder approach, an approach in which the diverse and valuable experiences of caregivers and children who have opted to move into the vanguard of AID need to be heard and appreciated.

## Appendix

Multimedia Appendix 1 Questionnaires for caregivers of children and adolescents with diabetes, using open-source automated insulin delivery.

Multimedia Appendix 2 Demographic and clinical characteristics of participants of the DIWHY study who have not responded to any of the open-ended questions.

## Abbreviations

AID: automated insulin delivery
CGM: continuous glucose monitor
CHERRIES: Checklist for Reporting Results of Internet E-Surveys
HbA1c: hemoglobin A1c
HCP: health care professional
OPEN: Outcomes of Patients’ Evidence with Novel, Do-it-Yourself Artificial Pancreas Technology
REDCap: Research Electronic Data Capture
T1D: type 1 diabetes
TIR: time in range
