# Supplementary material for: Emotional and Physical Health Impact in Children and Adolescents and Their Caregivers Using Open-source Automated Insulin Delivery: Qualitative Analysis of Lived Experiences
Source: J Med Internet Res. 2022 Jul 14;24(7):e37120. doi: 10.2196/37120 (PMC9335170; doi:10.2196/37120)
Supplement: Multimedia Appendix 2 [file jmir_v24i7e37120_app2.docx]

**Supplementary Table 1:** Demographic and clinical characteristics of participants of the DIWHY study who have not responded to any of the open-ended questions.

|  |  | **Children and Adolescents**  N=108 |
| --- | --- | --- |
| **Child’s gender** [n (%)] | Female | 55 (50.9) |
|  | Male | 53 (49.1) |
|  | Other | 0.0 (0) |
| **Child’s average age** [y±SD] |  | 9.6±3.6 |
| **Average duration of diabetes** [y ± SD] |  | 4.8±3.6 |
| **Average duration of open-source AID use**  [m ± SD] |  | 10.6±10.8 |
| **Region**  Country of residence   [n (%)] | ***Europe*** | ***75 (69.5)*** |
|  | Germany | 32 (29.6) |
|  | United Kingdom | 12 (11.1) |
|  | Czech Republic | 9 (8.3) |
|  | Bulgaria | 8 (7.4) |
|  | Others* | 14 (13.0) |
|  | ***North America*** | ***14 (13.0)*** |
|  | United States | 10 (9.3) |
|  | Canada | 4 (3.7) |
|  | ***Asia*** | ***9 (8.3)*** |
|  | South Korea | 9 (8.3) |
|  | ***Western Pacific*** | ***7 (6.5)*** |
|  | Australia | 7 (6.5) |
| **Caregiver’s education: highest completed**  [n (%)] | University Degree/Diploma | 57 (52.8) |
|  | Doctorate | 12 (11.1) |
|  | High School | 23 (21.3) |
|  | Other | 14 (13.0) |
| **Caregiver’s occupational status**  [n (%)] | Full-time | 60 (56.1) |
|  | Part-time | 37 (34.6) |
|  | Unemployed | 6 (5.6) |
|  | Other | 4 (3.8) |
| **Annual household income** [n (%)] | Under 20.000 USD | 14 (14.3) |
|  | 20.000 to 34.999 USD | 7 (7.1) |
|  | 35.000 to 49.999 USD | 15 (15.3) |
|  | 50.000 to 74.999 USD | 21 (21.4) |
|  | 75.000  to 99.999 USD | 12 (12.2) |
|  | Over 100.000 USD | 22 (22.4) |
|  | Not stated | 7 (7.1) |
